# Supplementary material for: Blood–brain barrier opening in Alzheimer’s disease using MR-guided focused ultrasound
Source: Nat Commun. 2018 Jul 25;9:2336. doi: 10.1038/s41467-018-04529-6 (PMC6060168; doi:10.1038/s41467-018-04529-6)
Supplement: Supplementary file 1 — Supplementary Information [file 41467_2018_4529_MOESM1_ESM.pdf]

Supplementary Information

Blood-Brain Barrier Opening in Alzheimer's Disease Using MR-guided Focused Ultrasound

Lipsman et al.

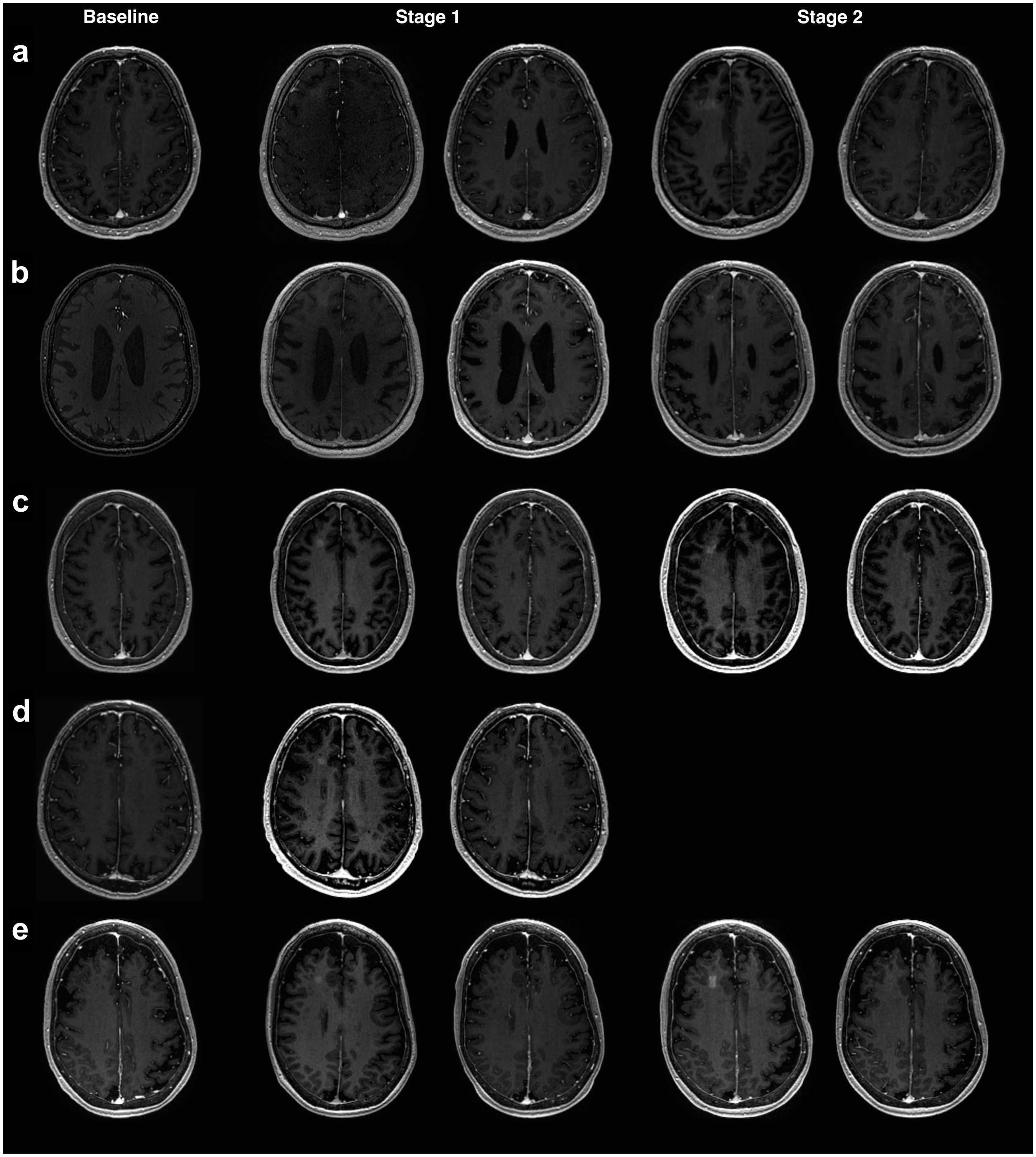

Supplementary Figure 1

Axial T1-weighted contrast enhanced MR images of patient 1 (a), 2 (b), 3 (c), 4, (d), and 5 (e) at key time points in the study. Images immediately after each sonication (left images of stage 1 and 2) shows contrast extravasation in the targeted volume in the right frontal lobe. Images obtained 24 hours after sonication (right images of stage 1 and 2) demonstrates resolution of enhancement.

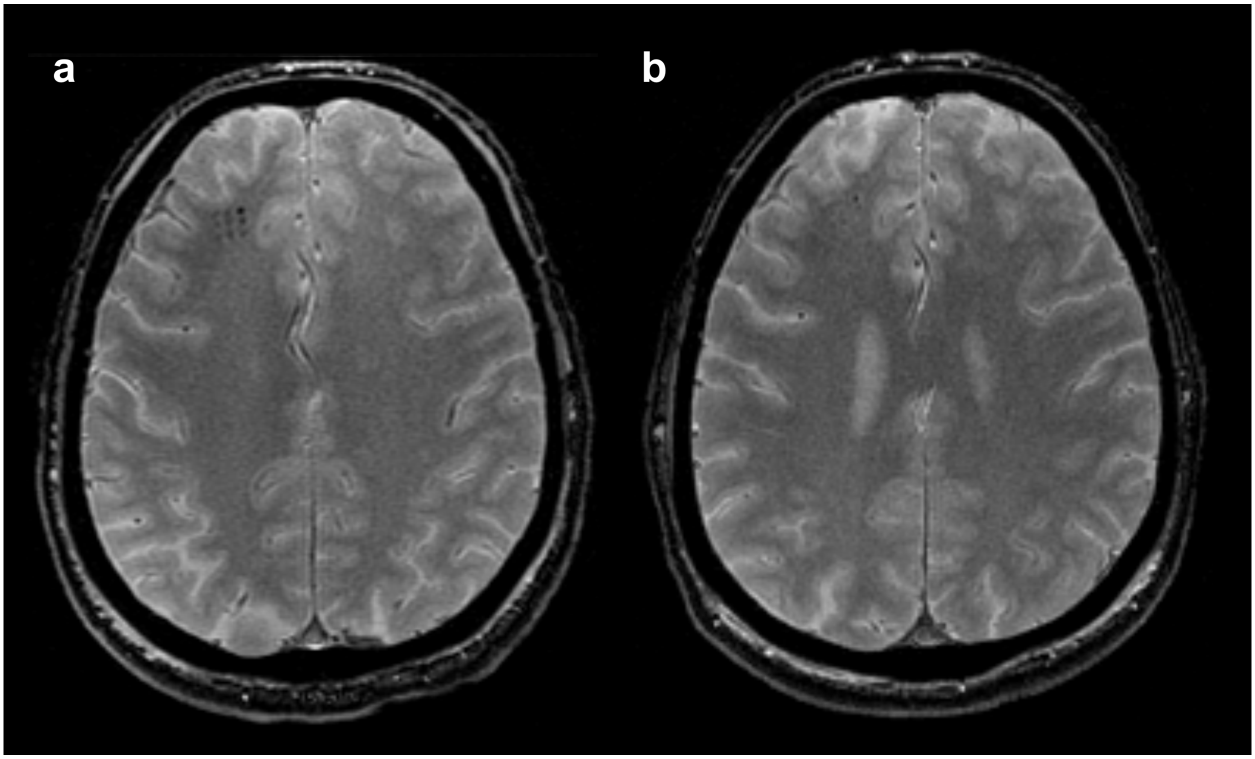

Supplementary Figure 2

(a) Axial T2*-weighted gradient echo MR scans of one patient show signal changes in the sonicated volume immediately after sonication. (b) 24 hours after sonication, the gradient echo signal changes are nearly resolved.

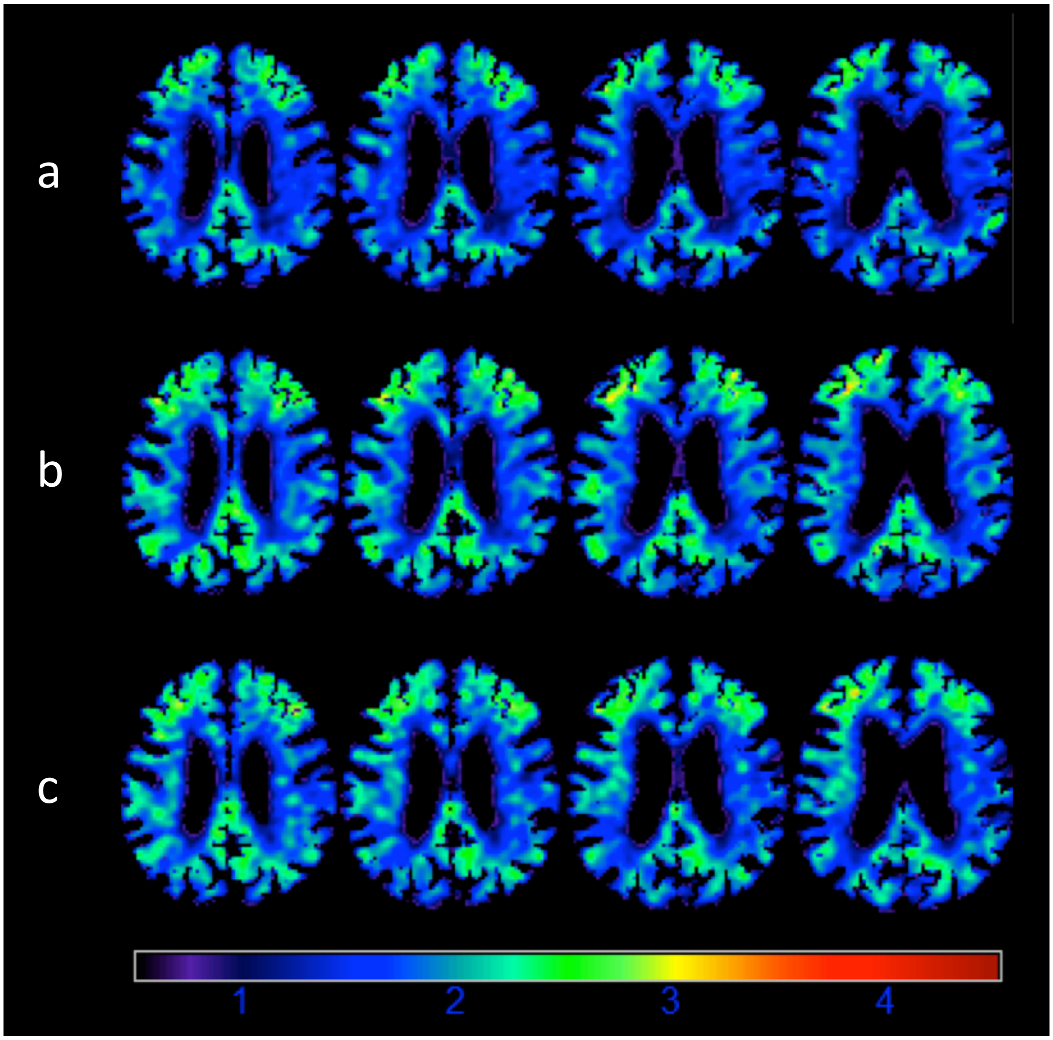

Supplementary Figure 3

Adjacent axial standardized uptake value ratio (SUVr) images for [^{18}F]-Florbetaben PET scans of patient 1 (a) at baseline, (b) one week after stage one, and (c) one week after stage two.

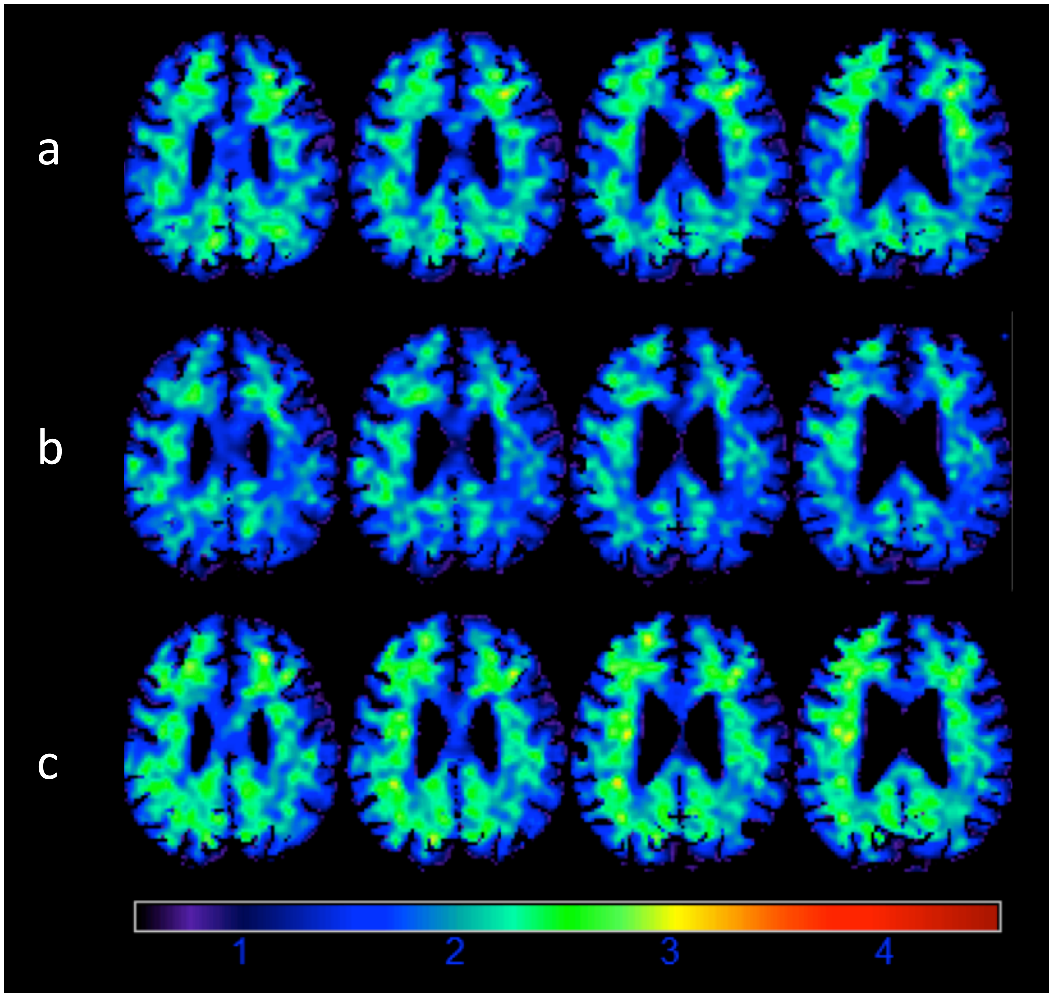

Supplementary Figure 4

Adjacent axial standardized uptake value ratio (SUVr) images for [^{18}F]-Florbetaben PET scans of patient 2 (a) at baseline, (b) one week after stage one, and (c) one week after stage two.

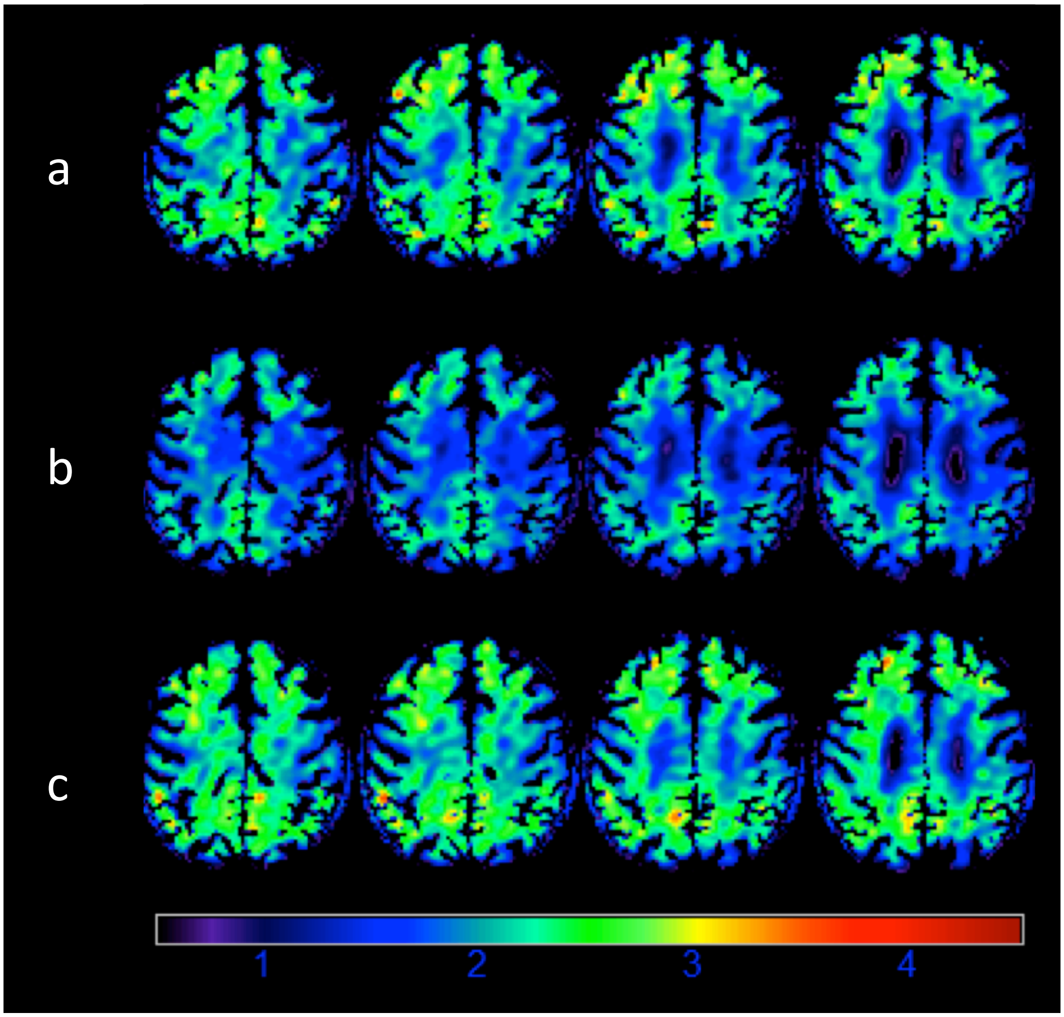

Supplementary Figure 5

Adjacent axial standardized uptake value ratio (SUVr) images for [^{18}F]-Florbetaben PET scans of patient 3 (a) at baseline, (b) one week after stage one, and (c) one week after stage two.

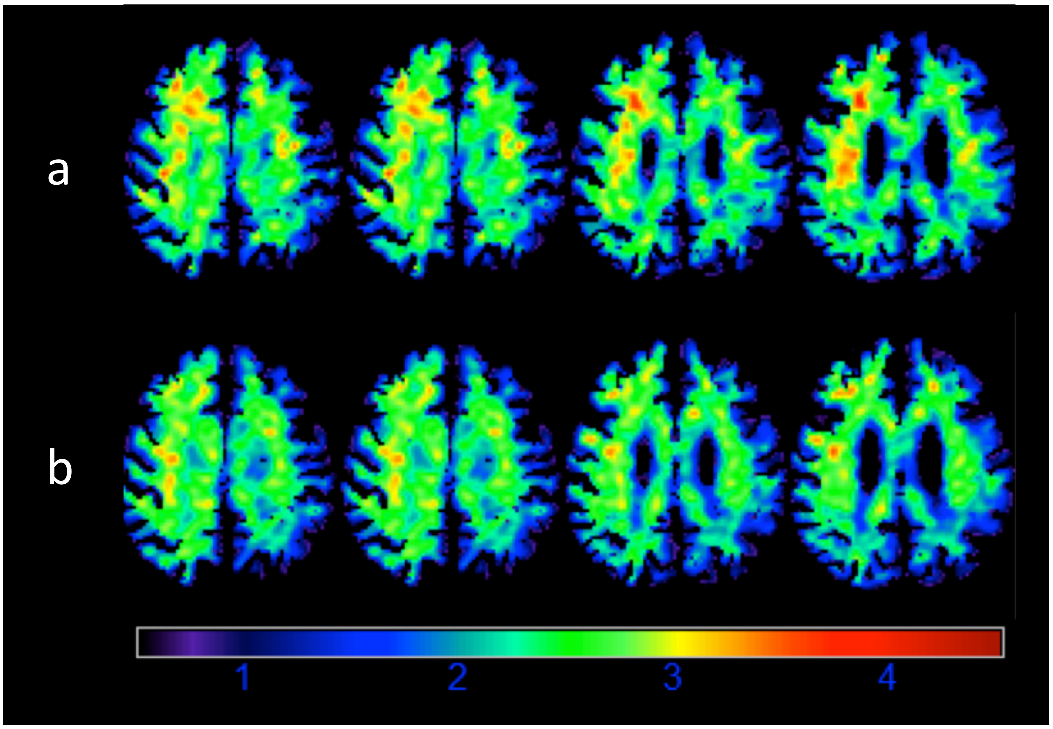

Supplementary Figure 6

Adjacent axial standardized uptake value ratio (SUVr) images for [^{18}F]-Florbetaben PET scans of patient 4 (a) at baseline and (b) one week after stage one.

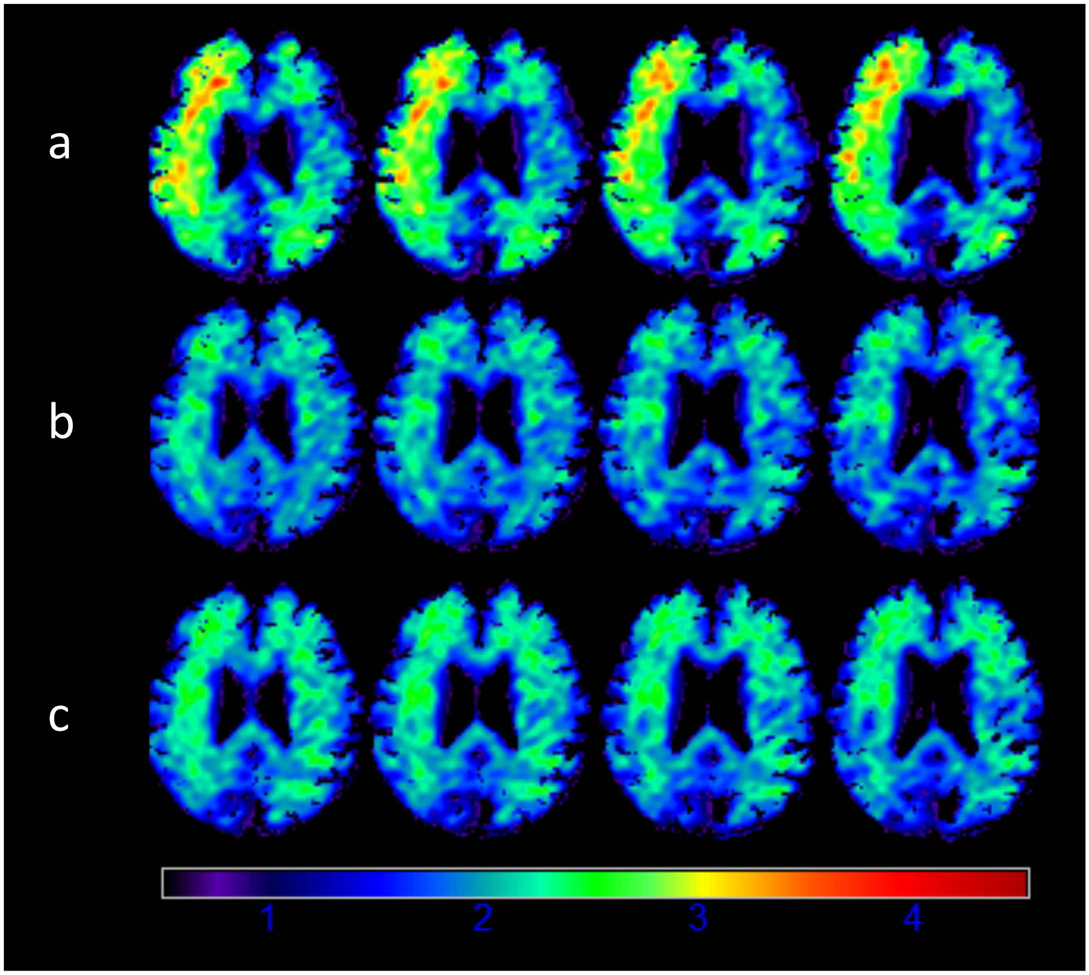

Supplementary Figure 7

Adjacent axial standardized uptake value ratio (SUVr) images for [^{18}F]-Florbetaben PET scans of patient 5 (a) at baseline, (b) one week after stage one, and (c) one week after stage two.

Supplementary Table 1. Sonication parameters

	Stage 1					Stage 2				
	Min power (W)	Max power (W)	Average sonication time (s)	Total no. of sonications	Total volume (mm^3)	Min power (W)	Max power (W)	Average sonication time (s)	Total no. of sonications	Total volume (mm^3)
**Patient 1**										
	7.5	7.5	50	2	567	3.0	3.5	50	6	1134
**Patient 2**										
	5.0	6.0	50	4	567	4.0	7.0	50	8	1134
**Patient 3**										
	3.0	3.0	50	4	175	2.5	3.5	50	8	350
**Patient 4**										
	2.5	3.0	50	6	175	-	-	-	-	-
**Patient 5**										
	3.5	3.5	50	2	175	3.5	5.0	50	8	350
**Mean (SD)**										
	4.3 (2.0)	4.6 (2.0)	50 (0)	3.6 (1.7)	331.8 (214.7)	3.3 (0.6)	4.5 (1.7)	50 (0)	7.5 (1.0)	742.0 (452.6)

Supplementary Table 2. Quantitative standardized uptake value ratio (SUVr) and percent change in SUVr in the regions of interest containing sonication volumes on [^{18}F]-florbetaben PET CT scans. SUVr was calculated by normalizing to the cerebellar gray matter.

	Stage 1			Stage 2		
	Baseline	7 days after	% Δ	Baseline	7 days after	% Δ
**Patient 1**						
	2.25	2.35	4.3	2.15	2.33	8.7
**Patient 2**						
	2.40	2.40	0.2	2.40	2.57	6.7
**Patient 3**						
	2.19	2.09	-4.7	2.32	2.35	1.6
**Patient 4**						
	3.01	2.75	-8.8	-	-	-
**Patient 5**						
	3.21	2.39	-25.6	3.10	2.51	-18.9
**Mean (SD)**						
	2.6 (0.5)	2.4 (0.2)	-6.9 (11.6)	2.5 (0.4)	2.4 (0.1)	-0.5 (12.6)

Supplementary Table 3. Percent change in standardized uptake value ratio (SUVr) in the regions of interest containing sonication volumes as well as the contralateral mirrored region on [^{18}F]-florbetaben PET CT scans. SUVr was calculated by normalizing to the cerebellar gray matter.

	**Stage 1**		**Stage 2**	
	% Δ in contralateral ROI	% Δ in treated ROI	% Δ in contralateral ROI	% Δ in treated ROI
**Patient 1**				
	4.2	4.3	0.1	8.7
**Patient 2**				
	-7.0	0.2	3.3	6.7
**Patient 3**				
	-7.3	-4.7	5.8	1.6
**Patient 4**				
	-7.2	-8.8	-	-
**Patient 5**				
	16.5	-25.6	6.1	-18.9
**Mean (SD)**				
	-0.2 (10.5)	-6.9 (11.6)	3.8 (2.8)	-0.5 (12.6)
